# Relationship between Sociodemographic and Health-Related Factors and Sedentary Time in Middle-Aged and Older Adults in Taiwan

**DOI:** 10.3390/medicina60030444

**Published:** 2024-03-08

**Authors:** Hung-Chin Huang, Shao-Hsi Chang, Xiaolin Yang

**Affiliations:** 1 Department of Physical Education and Sport Sciences, National Taiwan Normal University, Taipei 106308, Taiwan; 2 Likes, School of Health and Social Studies, Jamk University of Applied Sciences, 40100 Jyväskylä, Finland; xiaolin.yang@jamk.fi

**Keywords:** sedentary behavior, physical activity, TV viewing, sociodemographic variables, middle adulthood and old age, gender difference

## Abstract

*Background and Objectives*: This study aimed to investigate the associations between sociodemographic and health-related factors and sedentary time in middle-aged and older Taiwanese adults. *Materials and Methods*: A total of 1031 participants (460 men, 571 women; mean age 65.0 years ± 7.8 years; range 55 to 93 years) were randomly recruited from the National Computer Assessment Telephone Interview, Taiwan, in 2013. Sedentary time, TV viewing, physical activity, and sociodemographic factors were assessed through questionnaires. Body mass index was self-reported and calculated to evaluate obesity. In 2023, the associations between sedentary time and sociodemographic and health-related factors were analyzed using Pearson’s correlation, cross tabulation, and logistic regression and were stratified by gender. *Results*: Over 47% of participants reported spending more than 2 h watching TV, and more than 33% reported engaging in over 6 h of total sedentary activities. Men and women with insufficient physical activity had a higher probability of prolonged sedentary time than their physically active counterparts (*p* = 0.032 for men, *p* = 0.024 for women). Both men and women who spent more than 2 h watching TV daily were more likely to have high sedentary time compared to those with shorter TV viewing durations (both *p* < 0.001). Highly educated and unmarried women exhibited a higher likelihood of prolonged sedentary time than their less educated and married counterparts (*p* = 0.021 and *p* = 0.01, respectively). *Conclusions*: Sedentary time showed significant and positive associations with both insufficient physical activity and prolonged TV viewing in both genders. Additionally, significant associations were observed between sedentary time and high education and unmarried status in women. These findings emphasize the importance of implementing gender-specific approaches in future interventions and policy initiatives aimed at reducing sedentary behavior among middle-aged and older adults.

## 1. Introduction

In modern society, socioeconomic factors influence individuals’ health through rapid lifestyle changes. Sedentary lifestyles, which have become increasingly prevalent, often compromise people’s health [1]. Sedentary behavior increases the risks of chronic diseases, such as obesity, cardiovascular disease, and type 2 diabetes [2,3], and all-cause mortality [4]. In particular, older adults are at a high risk of sedentary behaviors because of the considerable increase in their leisure time after retirement and the aging of body functions [5,6]. Therefore, to reduce the health risks of older adults, interventions should be implemented to promote physical activity and modify sedentary lifestyles [5,6,7].

In Taiwan, the proportion of older adults aged >65 years has been increasing; the proportion of the older population is expected to reach 20% by 2025, leading to a super-aged society [8]. Regarding health-care resources, medical expenses for older adults account for 34.4% of national health-care costs, which is approximately 3.3 times higher than that for adults [9]. Therefore, from 2013 to 2023, research and policy development in Taiwan have focused on promoting the health of older individuals [10,11]. Research has confirmed that engaging in >150 min of physical activity every week promotes health [12]. Moreover, regular physical activity can mitigate the risks of obesity, cardiovascular disease, metabolic disease, hypertension, cancer, and mortality and reduce the number of clinic visits, which in turn reduces the health-care costs of older adults [7,12,13,14]. However, most studies have focused on the amount of physical activity in older adults [9,13,15,16,17,18]. Few studies have analyzed their sedentary behaviors.

Behavioral epidemiologists have begun to explore the health risks associated with sedentary behaviors [19]. Individuals spend most of their day engaging in sedentary behaviors, even if they meet the recommended standards for physical activity [20,21]. Sedentary behavior is defined as sitting or lying down for long periods during waking hours, which leads to sedentary lifestyles, low metabolic equivalent consumption, and inactivity [6]. Sedentary behaviors have an energy expenditure of 1.0–1.5 METs, which is lower than the energy expenditure for light physical activity (1.5–3 METs) [20,21]. Light physical activity includes sitting, driving, riding in a car, getting a haircut from someone else, watching sports, watching TV, doing crafts, talking, dating, participating in family gatherings, listening to music, reading, smoking, and watching movies in a theater [22]. Engaging in sedentary behavior for >6 h a day is associated with a high risk of type 2 diabetes, cardiovascular disease, hypertension, and cancer [2,23,24,25,26]. Accordingly, daily sedentary time of more than 6 h is an important cutoff point for investigating sedentary behaviors and health issues [27]. In a study of 20 countries, where the population was sedentary for >6 h a day, Taiwan ranked sixth, second only to Japan in Asia [23]. This finding indicates that the problem of sedentary behavior in Taiwan warrants further interventions and research efforts.

Sallis et al. [28] highlighted that traditional epidemiological explanations are limited for the elucidation of how individuals’ behaviors can lead to certain diseases. To date, some unhealthy behavioral patterns have been identified to be major causes of chronic disease, disability, and death. However, on the basis of the findings of Sallis et al. [28] and Owen et al. [1], they proposed an epidemiological framework for sedentary behavior after compiling studies on sedentary behaviors and health; the framework suggests that high-risk individuals who are likely to exhibit sedentary behaviors should be identified before the development of effective interventions for improving their behaviors. The recommendations have focused on children and young individuals [29,30], lacking specific guidance for middle-aged adults and the elderly. It is essential to enhance the evidence base concerning the correlation between sedentary behavior and health. This will be crucial in effectively formulating and implementing comprehensive sedentary behavior recommendations applicable to all age groups.

Of the various sedentary behaviors, watching TV remains the most prevalent in Western countries despite the proliferation of other electronic devices [31]. A systematic review has denoted that almost 60% of older adults report watching TV for more than 2 h [32]. Wijndaele et al. [33] reported the most significant gender difference, where 3.7% more females reported daily TV viewing exceeding 3.6 h. Some studies have reported associations between TV viewing and sociodemographic and health-related variables such as location, living alone, low education levels, part-time work, passive commuting, insufficient physical activity, and obesity [7,34]. The evidence has also highlighted associations between sedentary behavior and various factors, including education, age, employment status, gender, body mass index (BMI), income, smoking status, moderate-to-vigorous physical activity (MVPA), attitudes, and depressive symptoms/quality of life [35]. Noteworthy variations among specific sedentary behaviors were evident, contributing valuable insights for the interpretation of findings.

The aim of this study is to explore the associations of sociodemographic and health-related factors with sedentary time in middle-aged and older Taiwanese adults, stratified by gender. We hypothesized that sociodemographic factors would exhibit negative associations with sedentary time, whereas BMI would display a positive correlation with sedentary time. Specifically, we expected that sedentary behavior would be associated negatively with sociodemographic factors and positively with BMI, particularly in middle-aged and older women.

## 2. Materials and Methods

### 2.1. Study Population

The data for this study were collected from the National Telephone Database (Taiwan Trend Research Co., Ltd., Taipei, Taiwan) through the administration of the questionnaire (Computer Assessment Telephone Interview Version 1, Taipei, Taiwan) between June and July 2013 in Taiwan. To ensure consistency and reliability, a telephone interviewing company was contracted to employ two trained interviewers who were equipped to handle any potential issues and provide corresponding solutions during the interviews. The interview time was scheduled between 7 pm and 9 pm to minimize respondent unavailability due to work commitments. Participants completed a telephone interview during the same week in which they responded to the questionnaire regarding sedentary behavior, physical activity, and sociodemographic and health-related factors. For this study, a total of 1031 (96.5% of those invited) healthy adults aged 55 to 93 years were randomly selected from seven cities or regions in Taiwan: Taipei, New Taipei, Taichung, Tainan, Kaohsiung, Kinmen and Lianjiang, and Kinmen and Matsu. Ethics approval for the study was obtained from the ethics committee of the National Science and Technology Council (Approval No. 201309ES003) in 2013. All participants gave verbal consent before participating in the study.

### 2.2. Questionnaire

To determine sedentary behavior, participants were asked about the amount of time they had spent watching TV over the past week. Prolonged TV viewing was categorized as <2 h and ≥2 h per day for middle-aged and older adults [36,37]. The total sedentary time was assessed using the International Physical Activity Questionnaire (IPAQ) [38]. Participants reported their average sitting time during the past week, encompassing both weekdays and weekends. Subsequently, the average sitting time was then categorized as either <6 h per day or ≥6 h per day [27,39]. Physical activity was measured using the IPAQ-Computer Assessment Telephone Interview translated by Liou [38]. The Taiwan versions of IPAQ, including the self-administered long version, self-administered short version, and telephone interview short version, demonstrated high content validity with scores of 0.992, 0.994, and 0.980, respectively. In terms of intraclass correlation coefficients, consistency values for the English and Chinese versions were 0.945, 0.704, and 0.894, respectively. The IPAQ-Taiwan not only serves as a sensitive and precise tool but also displays the effectiveness of the applied cognitive aspect survey methodology in its development. Participants were queried about their physical activity levels over the past week, including the frequency and duration of physical activity engagement on weekdays and weekends. Specifically, durations of high-intensity exercise, moderate-intensity exercise, and walking were recorded. The total duration of physical activity was calculated by summing these individual durations. Physical activity levels were then categorized into two groups: those achieving ≥ 150 min per week and those achieving < 150 min per week [12].

Demographic information, including age, gender, and residential area (northern, central, southern, and eastern regions), was queried through a questionnaire. Given Taiwan’s legal retirement age of 65 years, age was divided into younger (<65 years) and older adult (≥65 years) groups. Marital status data categorized participants as either married (including those married and co-habiting) or unmarried (divorced and widowed). Work status was also queried, with participants classified as either full-time or other (part-time, housewives/husbands, unemployed, disabled, and retired). Participants self-reported their educational level, which was categorized as high school and above or junior high school and below based on completed years of schooling. Living arrangements were self-reported, with participants grouped as either living alone or living with family. Self-reported height and weight were used to calculate the body mass index (BMI) as weight (kg) divided by height (m^2^). The BMI was then classified according to the National Health Service of the Ministry of Health and Welfare guidelines as normal (BMI ≤ 24 kg/m^2^), overweight (BMI > 24–26.9 kg/m^2^), and obese (BMI ≥ 27 kg/m^2^) [40].

### 2.3. Statistical Analysis

Descriptive characteristics were expressed as the mean (SD) for continuous variables and as percentages for categorical variables. Gender differences in all variables were assessed using independent *t*-tests or *χ*^2^ tests, as appropriate. Pearson’s correlation coefficient (*r*) was employed to examine the correlation between sedentary time and socioeconomic and health-related variables. A cross-tabulation analysis was conducted to compare the frequencies of sedentary time levels with the levels of socioeconomic and health-related variables. Logistic regression analysis was employed to estimate the odds ratios (OR) with 95% confidence intervals (CI) for sedentary time (categorized). Both unadjusted and adjusted models were computed, adjusting for potential confounding variables including age, residential area, marital status, work status, education, living status, physical activity, TV viewing, and BMI (excluding its own variable). The analysis was performed using SPSS Statistics for Windows, version 23.0 (SPSS Inc., Chicago, IL, USA), in 2023. A two-sided *p* value of 0.05 was considered statistically significant.

## 3. Results

### 3.1. Characteristics of Participants

A total of 1031 middle-aged and older adults (460 men, 571 women) participated in this study. The age of participants ranged from 55 to 93 years with a mean age of 65.2 years ± 7.8 years. Table 1 presents the proportions of gender differences within each subgroup. Of the participants, 55.4% were female, 54.6% were aged under 65 years, 41.8% resided in the northern region, 84.6% were married, 69.1% did not work full-time, 52.7% had received education at the junior school and below level, 92.6% lived with family members, 60.6% were physically inactive for less than 150 min per week, and 52.6% watched TV for less than 2 h. Additionally, 33.1% reported spending more than 6 h per day sitting, 29.5% were overweight, and 14.6% were obese.

### 3.2. Intercorrelations between All Variables

The correlations between sedentary time and sociodemographic and health-related variables are shown in Table 2. There was a moderate positive association between sedentary time and TV viewing in both men (*r* = 0.36) and women (*r* = 0.37). Trivial associations were found between sedentary time and marital status in women (*r* = 0.13), as well as BMI in men (*r* = 0.09). Inverse associations were observed between sedentary time and physical activity in both genders (*r* = −0.17 for men, *r* = −0.13 for women), all of which were statistically significant (*p* < 0.05). Additionally, significant intercorrelations were found among certain variables.

### 3.3. Differences between Sedentary Time Level and Socioeconomic and Health-Related Variables

Participants who were married had a lower level of prolonged sedentary time than those who were unmarried (34.2% vs. 48.9%, *p* = 0.049 for men; 28.8% vs. 40.3%, *p* = 0.015 for women) (Table 3). Men and women with full-time work exhibited a lower level of prolonged sedentary time compared to those without full-time work (30.2% vs. 40.1%, *p* = 0.026 for men; 39.7% vs. 29.1%, *p* = 0.028 for women). Women who lived with family members demonstrated a lower level of prolonged sedentary time than those living alone (29.9% vs. 44.9%, *p* = 0.030). Participants who were physically active exhibited a lower level of prolonged sedentary time compared to their inactive counterparts (28.7% vs. 40.4%, *p* < 0.001 for men; 25.6% vs. 34.6%, *p* = 0.021 for women). Both men and women who spent less time watching TV had a lower level of prolonged sedentary time than those who watched more TV (23.3% vs. 51.2% for men; 19.3% vs. 42.8% for women, both *p* < 0.001).

### 3.4. Socioeconomic and Health-Related Determinants of Prolonged Sedentary Time

The results for the sociodemographic and health-related variables are presented in Table 4. Women were found to have increased odds of experiencing prolonged sedentary time than men (OR = 1.35, 95% CI = 1.04–1.75). Unmarried women had higher odds for prolonged sedentary compared to married women (OR = 1.38, 95% CI = 1.28–2.27). Additionally, women with higher levels of education exhibited increased odds of prolonged sedentary time than their less educated counterparts (OR = 1.63, 95% CI = 1.05–2.19). Compared to the physically active group, inactive participants showed higher odds of prolonged sedentary time (OR = 1.61, 95% CI = 1.09–2.14 for men; OR = 1.47, 95% CI = 1.11–2.87 for women). Participants who spent more time watching TV had higher odds of prolonged sedentary time than those who watched less TV (OR = 3.29, 95% CI = 2.17–4.99 for men; OR = 3.56, 95% CI = 2.36–5.37 for women). All associations remained significant after adjusting for covariates. No differences in BMI were observed between groups in either gender.

## 4. Discussion

We found that women tended to spend more time in sedentary activities than men. Both men and women who engaged in insufficient physical activity and had high TV viewing exhibited higher levels of sedentary time compared to those involved in MVPA and low TV viewing. Unmarried status and a lower education level were found to be significantly associated with increased sedentary time in women, even after adjusting for covariates. However, no significant associations were found between BMI and sedentary time in either gender. This suggests that certain socioeconomic factors directly influence sedentary time, particularly in middle-aged and older women.

The results revealed that men and women who participated in physical activity less than 150 min per week and watched TV for more than 2 h daily tended to spend more time in sedentary behavior than those who were physically active and spent less than 2 h on TV viewing. These results align with previous findings [7,27,41], indicating a consistent association between low physical activity levels and prolonged sedentary time across both genders. Mansoubi et al. [42] reviewed sedentary behavior associated with physical activity in adults. This implies that sedentary behavior may replace time allocated to light intensity activities. Therefore, interventions aimed at reducing sedentary behavior by promoting light activities hold significant potential for positively impacting public health.

Our results supported the hypothesis that women with higher levels of education and those who are unmarried tend to spend more time engaged in sedentary activities compared to their counterparts with lower education levels and marital status. These results align partly with previous research, which also identified a correlation between higher education and being unmarried with increased sedentary behavior levels [43,44]. An explanation for the gender differences in the relationship between socioeconomic status and sedentary behavior could be the higher prevalence of multiple physical conditions among women compared to men, particularly in older women [35]. In Taiwan, unmarried older women may find themselves with more leisure time due to their independent lifestyle and fewer family-related commitments, such as spending leisure time with family members, compared to their married counterparts. Consequently, the lifestyle of unmarried older women often involves prolonged periods of sedentary behavior, particularly spent watching TV. As a result, future interventions aimed at reducing sedentary behavior should prioritize unmarried, divorced, and widowed women.

Moreover, men and women often exhibit divergent cognitions and motivations regarding sedentary behavior: women tend to have negative intrinsic and extrinsic motivations towards such behaviors, while men typically possess positive intrinsic and extrinsic attitudes towards physical activity [45]. Consequently, women’s health consciousness may exert an additional influence on their decision-making processes regarding sedentary time. Another factor contributing to these differences could be the utilization of leisure time in Taiwan; for instance, Taiwanese women tend to spend an hour more on sedentary activities per day than men, potentially leading to a greater propensity for prolonged sitting. Future research could investigate gender-specific motives for sedentary behavior to verify whether factors such as motives or leisure time utilization significantly impact decision-making processes.

A study by Bauman et al. [23] conducted across 20 countries found that populations with higher education levels (>13 years) had significantly higher sedentary time compared to those with lower education levels (<13 years), consistent with our findings. However, Burton et al. [46] reported a positive correlation between prolonged TV time and lower education levels, which differs from our study. The results of Burton’s study also indicated that education level is associated with income level, whereas our study found that women with higher education levels were more likely to engage in daily physical activity. According to Burton et al. [46], highly educated older women, often with higher incomes, may be more inclined to purchase high-priced 3C products such as computers and tablets, leading to increased usage of these devices and subsequent sedentary behaviors such as reading books, newspapers, and magazines for extended periods.

Furthermore, Wallmann-Sperlich et al. [27], analyzing sedentary behaviors in 1986 German individuals, found that a higher education level was significantly associated with a daily sedentary time of >6 h in both genders, consistent with our results. Conversely, Hiddle et al. [3] displayed that Australian adults (aged ≥ 45 years) with lower education levels were more likely to engage in sedentary behaviors, contrasting with our findings. This discrepancy may be attributed to their study focusing on middle-aged adults, whereas future studies investigating the association between education level and sedentary time should emphasize older populations.

The literature on this topic presents varying results across different genders. In our study, we aimed to explore the relationship between sociodemographic factors and sedentary time in middle-aged and older men and women, with the goal of providing guidance to relevant authorities in designing direct and effective interventions for these population. Our findings revealed that middle-aged and older Taiwanese adults who watch TV for more than 2 h per day and engage in insufficient physical activity, at less than 150 min per week, are more likely to be sedentary for over 6 h per day compared to others. Notably, unmarried women and those with a higher education level were found to have the highest risk of being sedentary for over 6 h per day. Therefore, interventions targeting sedentary behaviors in these specific populations are urgently warranted.

This study did not find an association between sedentary time and BMI, which contrasts with findings from previous studies [35]. One possible explanation for this observation could be the measurement of BMI using self-report, which may compromise the accuracy of the results. Therefore, future research should consider employing objective methods for measuring BMI to provide more reliable insights into this relationship. Another potential explanation is that the criterion for BMI in Taiwan is based on the Asian standard levels, leading to variability in sedentary behavior markers and their respective cut-off points.

This study has several limitations that should be acknowledged. First, its cross-sectional design precludes the establishment of causal relationships between participants’ socioeconomic and health-related factors and sedentary time. Moreover, prolonged sedentary time may also influence socioeconomic and health-related factors in middle-aged and older adults, indicating the need for longitudinal research. Second, the study relied on a computerized telephone interviewing system to collect data on participants’ activities over the past week. While widely used, these questionnaires cannot verify participants’ sociodemographic status, levels of physical activity, TV viewing, and sedentary time, potentially introducing recall and social desirability biases. Third, the utilization of data collected in 2013 limits the contemporary relevance of the findings. Conducting similar research in Taiwan could serve as a baseline investigation, with subsequent analyses of new databases necessary to compare the findings. Lastly, the study only interpreted total sedentary time in terms of duration, without investigating the content of sedentary behavior. Future studies could benefit from utilizing accelerometer-derived assessments to gain a more nuanced understanding of sedentary behavior. Despite these limitations, the study provides valuable insights into the socioeconomic variables and sedentary time of middle-aged and older adults, aiding physical activity organizations and health centers in understanding the impact of sedentary time on the physical and mental health of this demographic group.

## 5. Conclusions

This study provides insight into the sociodemographic determinants of sedentary time in middle-aged and older adults in Taiwan. It highlights the important role that sociodemographic factors play in influencing sedentary time in this population. The findings suggest that certain factors may have gender-specific effects on prolonged sedentary time, indicating potential targets for interventions aimed at reducing excessive sedentary behavior. Future studies should aim to validate these findings using objective monitoring devices, which can offer a more comprehensive understanding of the impact of sedentary behavior on health outcomes. Additionally, efforts should be made to develop more effective strategies for reducing prolonged sitting and promoting active lifestyles. Such strategies could include environmental changes, improving sports facilities, promoting healthy diet, workplace interventions, and behavioral interventions. By implementing these multifaceted strategies, we can better address the health implications associated with sedentary behavior and contribute to the overall well-being and quality of life of middle-aged and older adults, with particular attention to the needs of women.

## Figures and Tables

**Table 1:** Characteristics of the study sample stratified by gender.

Variable		Total (*n* = 1031)		Men (*n* = 460)		Women (*n* = 571)	
		*n*	%	*n*	%	*n*	%
Age	<65 years	563	54.6	250	54.3	313	54.8
	≥65 years	468	45.4	210	45.7	258	45.2
Residential area	Northern	431	41.8	172	37.4	259	45.4
	Central	237	23	108	23.5	129	22.6
	Southern	311	30.2	151	32.8	160	28
	Eastern	52	5	29	6.3	23	4
Marital status	Married	872	84.6	410	89.1	462	80.9
	Unmarried	159	15.4	50	10.9	109	19.1
Work status	Full-time	319	30.9	197	42.8	122	21.4
	Other	712	69.1	263	57.2	449	78.6
Education level	≤Junior high school	543	52.7	201	43.7	342	59.9
	≥High school	488	47.3	259	56.3	229	40.1
Living status	Living alone	76	7.4	32	7	44	7.7
	Living with family	955	92.6	428	93	527	92.3
PA (min/week)	≥150 min	406	39.4	189	41.1	217	38
	<150 min	625	60.6	271	58.9	354	62
TV time (h/day)	<2 h	542	52.6	249	54.1	293	51.3
	≥2 h	489	47.4	211	45.9	278	48.7
ST (h/day)	<6 h	690	66.9	292	63.5	398	69.7
	≥6 h	341	33.1	168	36.5	173	30.3
BMI (kg/m^2^)	Normal (<24)	576	55.9	239	52	337	59.1
	Overweight (≤24–26.9)	304	29.5	157	34.1	147	25.7
	Obese (≥27)	151	14.6	64	13.9	87	15.2

Abbreviations: PA, physical activity; ST, sedentary time; BMI, body mass index; TV time, television viewing time.

**Table 2:** Pearson’s correlations between all variables stratified by gender.

Variable	1	2	3	4	5	6	7	8	9	10
1. ST (h/day)	-	0.080	−0.63	0.126 **	0.058	−0.053	−0.073	−0.126 **	0.369 **	0.062
2. Age	0.027	-	0.042	0.101 *	0.295 **	−0.144 **	−0.137 **	0.016	0.133 *	−0.103 *
3. Residential area	−0.019	0.022	-	0.001	0.025	−0.091 *	−0.080	−0.061	0.015	−0.044
4. Marital status	0.075	0.274 **	−0.043	-	0.148 **	−0.088	−0.342 **	−0.072	0.133 **	−0.098 *
5. Work status	−0.058	0.297 **	−0.078	0.128 **	-	−0.041	−0.037	−0.035	0.196 **	−0.035
6. Education level	0.035	−0.207 **	−0.079	−0.113 **	−0.177 **	-	0.098 *	−0.086	−0.154 **	0.020
7. Living status	−0.074	−0.172 **	−0.022	−0.348 **	−0.033	0.007	-	0.033	−0.160 **	0.008
8. PA (min/week)	−0.168 **	−0.020	−0.092 *	−0.042	0.003	−0.009	0.014	-	−0.005	−0.004
9. TV time (h/day)	0.357 **	0.057	−0.062	0.133 **	0.154 **	−0.056	−0.025	−0.106 *	-	0.087
10. BMI (kg/m^2^)	0.089 *	0.075	−0.042	0.062	0.090 *	−0.285 **	0.017	−0.004	0.224 **	-

Note: the upper matrix represents women, and the lower matrix represents men. Abbreviations: PA, physical activity; ST, sedentary time; BMI, body mass index; TV time, television viewing time. * *p* < 0.05, ** *p* < 0.01 (two-tailed).

**Table 3:** Cross-tabulation analysis of sedentary time level and socioeconomic and health-related variables stratified by gender.

Variable		Sedentary Time Level							
		Men (*n* = 460)				Women (*n* = 571)			
		<6 h (*n*, %)	≥6 h (*n*, %)	*χ* ^2^	*p* ^†^	<6 h (*n*, %)	≥6 h (*n*, %)	*χ* ^2^	*p* ^†^
Age	<65 years	167 (66.8)	83 (33.2)	1.265	0.261	237 (69.8)	76 (30.2)	0.335	0.563
	≥65 years	129 (61.8)	81 (38.2)			175 (67.6)	83 (32.4)		
Residential area	Northern	105 (61)	67 (39)	2.371	0.499	177 (68)	82 (32)	1.355	0.716
	Central	70 (64.7)	38 (35.3)			88 (67.9)	41 (32.1)		
	Southern	104 (68.9)	47 (31.1)			112 (69.4)	48 (30.6)		
	Eastern	18 (60.7)	11 (39.3)			19 (79.2)	4 (20.8)		
Marital status	Married	270 (65.8)	140 (34.2)	3.836	0.049	329 (71.2)	133 (28.8)	5.932	0.015
	Unmarried	26 (51.1)	24 (48.9)			66 (59.7)	43 (40.3)		
Work status	Full-time	138 (69.8)	59 (30.2)	4.950	0.026	74 (60.3)	48 (39.7)	4.849	0.028
	Other	158 (59.9)	105 (40.1)			319 (70.9)	130 (29.1)		
Education level	≤Junior high school	131 (64.8)	70 (35.2)	0.067	0.796	239 (69.7)	103 (30.3)	0.982	0.322
	≥High school	165 (63.6)	94 (36.4)			149 (64.7)	80 (35.3)		
Living status	Living alone	14 (48.4)	18 (51.6)	3.714	0.054	25 (55.1)	19 (44.9)	4.700	0.030
	Living with family	281 (65.5)	147 (34.5)			370 (70.1)	157 (29.9)		
PA (min/week)	≥150 min	135 (71.3)	54 (28.7)	6.851	0.009	162 (74.4)	55 (25.6)	5.365	0.021
	<150 min	162 (59.6)	109 (40.4)			232 (65.4)	122 (34.6)		
TV time (h/day)	<2 h	191 (76.7)	58 (23.3)	38.35	<0.001	237 (80.7)	56 (19.3)	37.129	<0.001
	≥2 h	103 (48.8)	108 (51.2)			160 (57.2)	118 (42.8)		
BMI (kg/m^2^)	Normal	158 (65.9)	81 (34.1)	1.038	0.595	240 (71.1)	97 (28.9)	4.406	0.110
	Overweight	102 (64.4)	55 (35.6)			102 (68.9)	45 (31.1)		
	Obese	38 (59.1)	26 (40.9)			53 (59.8)	34 (40.2)		

^†^ The *p* value for group difference (Pearson’s chi-square test). Abbreviations: PA, physical activity; BMI, body mass index; TV time, television viewing time.

**Table 4:** Regression coefficient of sociodemographic and health-related factors on sedentary time levels stratified by gender.

Variable	High ST (≥6 h) in Men ^†^				High ST (≥6 h) in Women ^†^			
	Unadjusted		Adjusted ^‡^		Unadjusted		Adjusted ^‡^	
	OR (95% CI)	*p*	OR (95% CI)	*p*	OR (95% CI)	*p*	OR (95% CI)	*p*
Age								
<65 years	1.00 (ref.)		1.00 (ref.)		1.00 (ref.)		1.00 (ref.)	
≥65 years	1.38 (0.91–2.09)	0.137	1.22 (0.79–1.89)	0.362	0.96 (0.65–1.43)	0.857	1.08 (0.71–1.63)	0.692
Residential area								
Northern	1.00 (ref.)		1.00 (ref.)		1.00 (ref.)		1.00 (ref.)	
Central	0.87 (0.51–1.49)	0.622	0.86 (0.69–1.89)	0.165	1.28 (0.79–2.08)	0.322	0.74 (0.72–1.66)	0.215
Southern	0.86 (0.52–1.37)	0.509	0.85 (0.38–1.17)	0.331	0.78 (0.49–1.26)	0.316	1.22 (0.89–2.13)	0.459
Eastern	1.04 (0.44–2.48)	0.934	0.79 (0.52–1.22)	0.844	0.43 (0.14–1.27)	0.134	0.44 (0.27–1.64)	0.778
Marital status								
Married	1.00 (ref.)		1.00 (ref.)		1.00 (ref.)		1.00 (ref.)	
Unmarried	1.20 (0.60–2.38)	0.617	1.44 (0.69–3.01)	0.332	2.21 (1.33–3.68)	<0.001	1.38 (1.28–2.27)	0.010
Work status								
Full-time	1.00 (ref.)		1.00 (ref.)		1.00 (ref.)		1.00 (ref.)	
Other	1.02 (0.67–1.54)	0.935	0.86 (0.55–1.34)	0.516	0.67 (0.41–1.08)	0.109	0.72 (0.38–1.81)	0.176
Education level								
≤Junior high school	1.00 (ref.)		1.00 (ref.)		1.00 (ref.)		1.00 (ref.)	
≥High school	1.23 (0.81–1.86)	0.333	1.09 (0.70–1.70)	0.693	1.77 (1.18–2.66)	0.012	1.63 (1.05–2.19)	0.021
Living status								
Living alone	1.00 (ref.)		1.00 (ref.)		1.00 (ref.)		1.00 (ref.)	
Living with family	0.84 (0.36–1.97)	0.704	0.55 (0.23–1.32)	0.188	0.71 (0.34–1.48)	0.367	0.49 (0.23–1.01)	0.545
PA								
≥150 min	1.00 (ref.)		1.00 (ref.)		1.00 (ref.)		1.00 (ref.)	
<150 min	1.80 (1.18–2.75)	0.010	1.61 (1.09–2.14)	0.032	1.52 (1.01–2.28)	0.043	1.47 (1.11–2.87)	0.024
TV time								
<2 h	1.00 (ref.)		1.00 (ref.)		1.00 (ref.)		1.00 (ref.)	
≥2 h	3.21 (2.14–4.81)	<0.001	3.29 (2.17–4.99)	<0.001	3.53 (2.35–5.31)	<0.001	3.56 (2.36–5.37)	<0.001
BMI (kg/m^2^)								
Normal	1.00 (ref.)		1.00 (ref.)		1.00 (ref.)		1.00 (ref.)	
Overweight	0.97 (0.62–1.52)	0.891	1.10 (0.83–1.47)	0.487	0.97 (0.60–1.54)	0.885	1.16 (0.89–1.50)	0.266
Obese	1.29 (0.70–2.35)	0.417	1.31 (0.76–2.44)	0.314	1.51 (0.87–2.61)	0.146	1.22 (0.53–1.23)	0.493

^†^ The regression models with low sedentary time (<6 h) of men and women coded as a reference level. ^‡^ Parameters were adjusted for age, residential area, marital status, work status, education, living status, physical activity, TV time, and BMI (except for each one), respectively. Abbreviations: PA, physical activity; ST, sedentary time; BMI, body mass index; TV time, television viewing time.

## Data Availability

The data presented in this study are available on request from the corresponding author. The data are not publicly available due to privacy.
